# Microfluidic Systems for Cancer Diagnosis and Applications

**DOI:** 10.3390/mi12111349

**Published:** 2021-10-31

**Authors:** Semra Akgönüllü, Monireh Bakhshpour, Ayşe Kevser Pişkin, Adil Denizli

**Affiliations:** 1 Department of Chemistry, Faculty of Science, Hacettepe University, Ankara 06800, Turkey; semraakgonullu@hacettepe.edu.tr (S.A.); b.monir@hacettepe.edu.tr (M.B.); 2 Department of Medical Biology, Faculty of Medicine, Lokman Hekim University, Ankara 06230, Turkey; akpiskin@gmail.com

**Keywords:** biomaterials, microfluidics, single cell analysis, cancer diagnosis

## Abstract

Microfluidic devices have led to novel biological advances through the improvement of micro systems that can mimic and measure. Microsystems easily handle sub-microliter volumes, obviously with guidance presumably through laminated fluid flows. Microfluidic systems have production methods that do not need expert engineering, away from a centralized laboratory, and can implement basic and point of care analysis, and this has attracted attention to their widespread dissemination and adaptation to specific biological issues. The general use of microfluidic tools in clinical settings can be seen in pregnancy tests and diabetic control, but recently microfluidic platforms have become a key novel technology for cancer diagnostics. Cancer is a heterogeneous group of diseases that needs a multimodal paradigm to diagnose, manage, and treat. Using advanced technologies can enable this, providing better diagnosis and treatment for cancer patients. Microfluidic tools have evolved as a promising tool in the field of cancer such as detection of a single cancer cell, liquid biopsy, drug screening modeling angiogenesis, and metastasis detection. This review summarizes the need for the low-abundant blood and serum cancer diagnosis with microfluidic tools and the progress that has been followed to develop integrated microfluidic platforms for this application in the last few years.

## 1. Introduction

Cancer remains one of the most common causes of mortality in the world [1,2,3,4,5]. It is the paramount human health problem. Cancer is still one of the main threatening diseases in the world, accounting for approximately 10 million deaths in 2020 [6,7,8]. The effects of global climate changes on geographies and changing dietary habits are accepted as some of the factors that lead to cancer. Although the exact cause of cancer is not clear, it is known that cancer cells multiply very quickly. Therefore, the detection of cancer cells is of great significance for early diagnosis. Many current imagining technologies have been employed to define and locate the nature of the tumor mass such as biopsies, positron emission tomography (PET), diagnostic magnetic resonance imaging (MRI), and computed tomography (CT) [9]. However, these methods are generally very expensive and have some drawbacks [10,11,12,13]. Sometimes, these devices can cause adverse effects on cancer patients [14,15]. The current imaging techniques are applied for therapy follow-up, exposing the patients to a high dose of radiation that may be bad for healthy tissues and cells, causing long-term effects [16,17]. Given that cancer is the most common cause of disease-related mortality and the need to limit the application of current unpleasant, invasive, and inappropriate clinical diagnostic methods, there is an increasing requirement for quick, non-invasive, even direct techniques for cancer diagnosis [18,19,20,21]. A diagnostic procedure that can rapidly and accurately analyze changes for cancer biomarkers in biological fluids will widely simplify cancer monitoring, diagnosis, therapy, and prognosis [22,23,24]. Breast, lung, colorectal, and cervix cancers are the most common in women while prostate, lung, stomach, colorectal, and liver cancer are the most widespread kinds of cancer in men [25]. For this reason, sensing systems are the solution for such limitations and are utilized in the diagnosis of different cancers including prostate, lung, breast, ovarian, liver, and other cancers. Their low-cost and fast processing make them very advantageous [26,27,28,29,30].

Microfluidic is a term that refers to engineered manipulation of fluid flow that is geometrically constrained to micro-sized objects [31,32]. Microfluidic systems were improved with the aim to develop some characteristics of conventional analytical methodologies, by making smaller and increasing the automation of the analytical systems. Microfluidic tools handle and manipulate fluids at a submillimeter scale from 1 to 1000 μm, with typical internal volumes of microliters to picolitres [33,34,35]. These small length scales provide several advantages such as high sensitivity, low-consumption of reagents, laminar flow, cost effectiveness, and high spatiotemporal resolution [36,37]. This technology has been reported in the last decades for various applications of biological and medicinal antimicrobial screening [38], cell culture [39], drug release systems design [40], DNA amplification [41], nanomaterials synthesis [42] and also point-of-care (POC) systems development [43,44]. Microfluidic tools can be used in subject areas from biological analysis and chemical synthesis to optical and information technology [45,46,47,48,49,50,51]. A miniaturized fluidic channel provides a potential tool for experimental biological investigation [52]. The development of micro-fabrication techniques for the semiconductor industry inspired microscale instruments for biological investigation (to route molecules and cells). As micro- and nano-generation techniques have improved, so have the microfluidic vehicles that are particularly fabricated for biological research. For instance, the improvement of techniques for micro instrument prototyping eliminated the requirement for lengthy production processes in semiconductor clean-room facilities, thereby making the creation of tools quick and possible in biological laboratories [53]. Microfluidic platforms also allow the combination of several steps in single automated platforms, lowering time spent and cost on analysis, in comparison with traditional techniques [54].

Microfluidic tools have become important models for cancer investigation over the past decade [25]. This technology has indicated a great potential for the design of novel methodologies for cancer diagnosis, monitoring, treatment, and disease follow-up. A variety of in vivo, ex vivo, and in vitro experimental models have been traditionally used to discover therapeutic targets and test novel drugs for cancer [55]. As a result, microfluidic tools have the potential as a novel platform to carry out cancer research, monitoring and diagnosis [36]. Therefore, sensitive detection and selective identification of circulating cancer biomarkers is a crucial step in cancer clinical diagnostics.

## 2. Microfluidic Technologies

The contribution of biosensor techniques to existing health systems is very important [56,57,58]. As the world population grows and ages, the healthcare industry aims to replace existing biosensor devices with portable alternatives that may be applied in homecare and doctor’s offices. Nowadays, computing power and technology has laid the foundation for portable biosensing tools that can radically change the efficacy, quality and, mode of clinical trials [59].

Microfluidics as an expanded technology is used with small channels, ranging from tens to hundreds of micrometers in height or width for handling small fluid volumes. Microfluidics makes use of its characteristic small size and characteristics of fluids in microchannels, such as laminar flow [46]. Overall, microfluidic assays represent a dramatic improvement in physiological relevance over other in vitro models because they allow precise control of the cellular, physical, and biochemical microenvironment. The microfluidic technology seems almost too good, and it presents so many advantages and very few disadvantages in its analytical applications. Therefore, these tools have been broadly used. The area of microfluidics has four branches: molecular analysis, microelectronics, biodefence, and molecular biology [46]. Microfluidics systems are used in many areas such as clinical diagnostics [60], anticancer drug studies [5], drug discoveries [61], protein crystallization [62], point of care diagnostics [63], single cell analyses [64], cell-to-cell interaction [65].

Microfluidic tools that need only a drop of a sample, provide important advantages over conventional instruments via lab-chip approaches to detect biological target analytes from small volumes of biological samples in a relatively short analysis time [46,66]. These techniques can further develop the simplicity of the assay, hence potentially accelerate their reach to the point of care settings [59,67,68,69]. The microfluidic chips have properties such as quick and effective analysis, low consumption, and miniaturization [70].

To advance novel therapeutic approaches at the age of personal medicine, it is significant to comprehend the functional characteristics of proteins, ribonucleic acid (RNA), and deoxyribonucleic (DNA) at the sole-molecule level in personal cancer cells. Microfluidic tools have emerged as a recent technology which provides advantages in single molecules and single cells analysis with perfect sensitivity [70,71]. Here, we present and highlight some recent works that used microfluidic tools for early cancer diagnostics and applications. We have also examined current assays and discussed the development of new microfluidic technologies for cancer biomarker detection.

## 3. Microfluidic Tools for Cancer Diagnosis

The technology of microfluidic systems provides a significant possibility for using the sensor devices for a wide range of applications such as clinical diagnosis, biological detection, and environment or wastewater monitoring [72,73,74]. Microfluidic technology has been used in the past decade as a noteworthy device for cancer research [25,55]. Microfluidic technologies combined with biosensors are more sensitive and precise in the detection of cancer biomarkers than classic platforms [75,76]. Here, the latest progress trends of microfluidic systems as novel detection instruments and their use in cancer research, diagnosis and therapy is reviewed. This report also examines the prospects of microfluidic sensor systems, and the combination of these systems with different materials.

Nguyen et al. designed an electrical cell-impedance sensing that integrated with microfluidic chip using monitoring single cancer cell migration in 3-D matrixes. Kinetic information was provided by these integrated microfluidic systems about cell migration. The author developed an electrical cell integrated microfluidic system for detecting a single cancer cell migration. The microfluidics techniques can efficiently capture cancer cells sequentially without the necessity of physical interaction to off chip pneumatics. The detection of single cancer cell migration was shown successfully. Nguyen and coworkers demonstrated real time detection of MDA MB 231 cells in the initial stage of migration in metastases. The designed chip showed a selective, real-time and rapid detection of the migratory properties of cancer cells at the single-cell level. This technique can successfully be applied to cancer cell research as a novel device [77]. 

**Figure 1:**
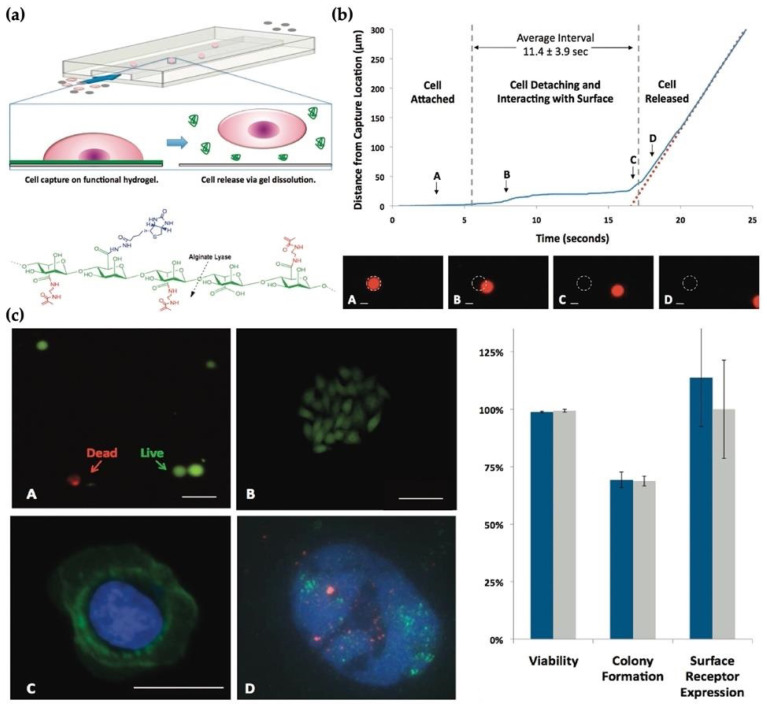
(**a**) Sacrificial hydrogel coatings microfluidic devices; (**b**) The gentle nature of the release process as the cell starts; (**c**) released cells for (**A**) viability using a fluorescent LIVE (green)/DEAD (red) assay and (**B**) colony formation, (**C**) immunostaining of cell surface receptors, (**D**) FISH (fluorescence in situ hybridization) analysis in a released HER2 (green probe) amplified breast cancer cell; the control probe (red) [78].

Shah et al. implemented a smart technology for cell capturing using a biopolymer system to recover cells from microfluidic devices [78]. The specific cell isolation is obtained using an affinity-based microfluidic system from a complex material such as tumor cells in whole blood. The innocuous recovery of cells that attach on the surface of the microfluid system is an important parameter in this technology. The authors demonstrated a bio-functional hydrogel coating for microfluidic chips as seen in Figure 1a. They showed that this platform is more stable in a broad diversity of physiological solutions. Also, they used this system for the capture and release of epithelial cell adhesion molecule (EpCAM) expressing cancer cells. Prostate cancer accounts for almost 10% of all cancer-related deaths and it is the third most common cancer in men [79]. Prostate cancer and breast cancer cell lines were employed in this study. No significant effect was found on proliferative potential and cell viability, and they recovered cells during capture and release process. In addition, they compared this with FISH (fluorescence in situ hybridization) analysis and downstream immunostaining. A typical captured cell was used to deliver the cell release procedure and was imaged during the release. Also, the position of the cell was tracked over time. The degradation of gel, capturing the cell, detaching, and slowly moving was given in Figure 1b. The release efficiency of this study was found to be 99 ± 1%. Also, the release efficiency was obtained as 69.3 ± 3.4% by diluting released cells in a culture medium (Figure 1c) [78].

**Figure 2:**
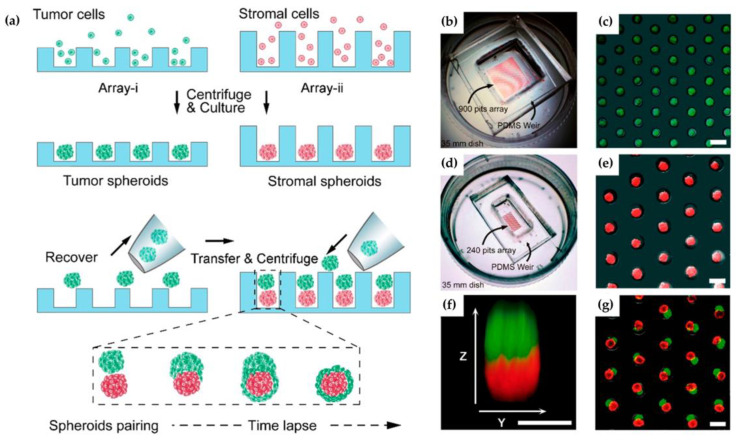
(**a**) The microfluidic micro well array-based spheroids pairing; (**b**–**e**) representative pictures of chips; (**f**) tumor−stroma pairing with green and red fluorescence; and, (**g**) image of heterotypic cell spheroid pairs [80].

The primary tumors through metastasis can cause the main challenge in treating cancer and most cancer mortalities. In another study, Zhao et al. prepared microfluidic systems for the 3-D metastasis study of the tumor and stromal cell spheroids merging and pairing. Since the interaction between tumor and stroma naturally plays the main role, 3-D cell culture may provide an efficient method to simulate tumor microenvironment in vivo. However, the 3-D culture was ineffective at showing the definite existential heterogeneous pairing. In this study, the author carried out a microfluidic system to dissect an in vitro process of cell spheroids-based tumor and stromal interaction. This device can properly show one-to-one pairing tumor-fibroblast spheroid for investigating 3-D tumor irruption [80]. The methodology of this study is summarized in Figure 2a. There, two independent microfluidic chips were designed. Figure 2b,c shows the generating tumor cell aggregates within 24 h for the first chip. Figure 2d,e also demonstrate both functions of the second chip.

**Figure 3:**
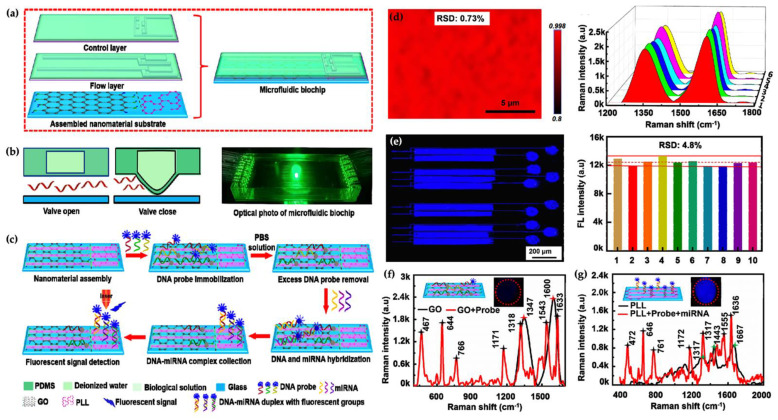
İllustration of the microfluidic chip; and the Raman mapping and representative (**a**–**c**) was also used to indicate all areas of interest and were assembled with graphene oxide (GO). (**d**–**g**) shows the fluorescence mapping images of the detection chamber [81].

In recent years, enormous efforts in sensing techniques of various miRNA have provided developed performance of miRNA sensor platforms. To develop the detection throughput and performance microfluidic technology have been combined into diverse sensing systems. The microfluidic-based sensing tools have been employed to obtain an attomolar(aM)-level multiple-miRNA sensing platform with high performance. Chu et al. developed a nanomaterial-based microfluidic chip for ultra-sensitive detection of microRNA (miRNA) at attomole levels for use in cancer diagnosis. Real-time and early detection plays an important role in disease management. Over the last decade, miRNA detection for non-invasive early diagnosis is of great significance in not only diagnosis but also for the subsequent planning of the medical treatment regime. Sensitive, fast, real-time, and economic miRNA detection devices are essential to sense miRNA at low concentrations in biological and clinical diagnosis studies. In this study, five representative miRNA biomarkers (miR-125, miR-126, miR-191, miR-155, and miR-21) in breast cancer were chosen to indicate the microfluidic biochip’s performance. The author reported a linear range detection between 1.0 aM and 10 nM and showed a 0.146 aM limit of detection value without any amplification in 35 min detection time using 2.0 μL sample volume. The five miRNA samples were analyzed and reported from healthy humans and breast cancer patients. These results demonstrated the great capacity for early detection of cancer [81]. Illustration of the microfluidic chip is shown in Figure 3a–c. The Raman mapping was also used to indicate all areas of interest and were assembled with graphene oxide (GO) as a novel nanomaterial. Figure 3d–g shows the fluorescence mapping images of the detection chamber.

Otieno et al. designed a microfluidic immunoarray platform for multiplex and sensitive detection of parathyroid hormone-related peptides (PTHrP) in cancer diagnostics. PTHrP is known as the important initiative agent of HHM (humoral hypercalcemia of malignancy). The paraneoplastic PTHrP has also been implicated in the metastasis of cancers. Generally multiplexed and ultrasensitive detection by immunoassay is a major challenge in this area. In this study, authors prepared an ultrasensitive multiplexed peptide assay to detect PTHrP 1-173. They used this system with a chamber for real-time capture of the peptides with magnetic beads located with enzyme labels and peptide-specific antibodies. They reported 150 aM limit of the detection value by using 5 μL serum sample in 30 min [82]. Zhou et al. developed a microfluidic featuring platform for characterization and electrical impedance of human cancer cells. They designed a microfluidic system to characterize the electrical and mechanical properties of individual cells at the same time in a high-throughput manner. Also, the characterized deformability of breast cancer cells (MCF-7) based on the passage time required for an individual cell to pass through a constriction smaller than the cell size [83]. Circulating tumor cells as an indicator are usually used for management of cancer treatment. However, this method presents some disadvantages such as no reliable capturing, isolation, and enumeration of cells. In view of this, recently many microfluidic systems for this purpose have been developed. Ren and their colleagues prepared novel multiple rows and size-based detection of circulating tumor cells. They used a microfluidic platform for capturing LNCaP-C4-2 prostate cancer cells. In this study, they optimized the capture ratio and limit on all of the rows and showed that five/six rows of micro constriction channels for trapping chambers were needed to achieve >95% capturing ratio [84].

High rates of glycolysis in tumors have been associated with tumor recurrence, cancer metastasis, and poor outcomes. The single cells that exhibition high glycolysis are specific targets for therapy. Zielke et al. used a microfluidic device for the isolation of cancer cell subpopulations based on single cells glycolysis. Glycolysis with a high rate has been correlated with tumor recurrence, cancer metastasis, and poor outcomes. Therefore, a single cell is used as specific target in the therapy process. The study of this single cell needs an efficient device for sensitive isolation of the cell. The author utilized a droplet microfluidic platform to use in glycolysis-based isolation of cell subpopulations without any labeling. This designed platform was shown to be a robust and easy way of single cell isolation [85].

Malhotra et al. designed an ultrasensitive electrochemical microfluidic array optimized for the detection of a four-protein panel of biomarker proteins. The validation of a four-protein panel was done with oral cancer diagnostics. They reported 5−50 fg·m/L range detection and reported 6 (IL-6), IL-8 ultralow detection for a vascular endothelial growth factor, and vascular endothelial growth factor-C in serum. Off-line protein capture was used by magnetic beads carrying ∼100,000 antibodies and 400,000 enzyme labels for achieving high sensitivity in 50 min assays. The magnetic beads were separated after the capture of the proteins. Then proteins were injected into the array for selective capture by antibodies on eight nanostructured sensors. The easily fabricated and low-cost immunoarray can be provided with an easy serum test for diagnosis and also personalized therapy of oral cancer. They demonstrated a device that can be adapted to clinical diagnostics of cancers [86].

In another study, the mechanical phenotype of the human colon adenocarcinoma cell was investigated. This type of cancer is known to be a heterogeneous cell line with both multipotency and self-renewal abilities. This cancer stem-like cell can be the originator of all tumor cells. The authors designed a combined microchannel with an ionic current system to detection of high heterogeneity of cell deformability in the population of HT29 cells. They reported that the development of non-destructive identification and collection techniques for cancer stem-like cells has not only been for cancer diagnosis and prognosis but also for the discovery of a new treatment for cancer [87].

Lung cancer is one of the leading causes of cancer-related high mortality globally. Early diagnosis in this cancer is related with a favorable prognosis, but approximately 57% of lung cancer patients are diagnosed at the advanced stages [88,89]. In a novel study, a microfluidic tool was developed and improved their purity through fluid dynamics for easily isolated cancer cells. This high-throughput and label-free system continuously isolates cancer cells and other unrelated molecules from pleural effusion. Most of the background cells that affect interpretation are flushed to outlets 1 to 3, and cancer cells are hydrodynamically concentrated to outlet 4, with 90% of lung cancer cells flowing to this outlet. In lung cancer, malignant pleural effusion is a possible complication of the disease. After processing, the purity of cancer cells identified in pleural effusion by CD45 and epithelial cell adhesion molecule antibodies in flow cytometry will be increased by 6 to 24 times. This microfluidic platform showed low cost and rapid processing to rapid and direct clinical diagnosis [90].

**Figure 4:**
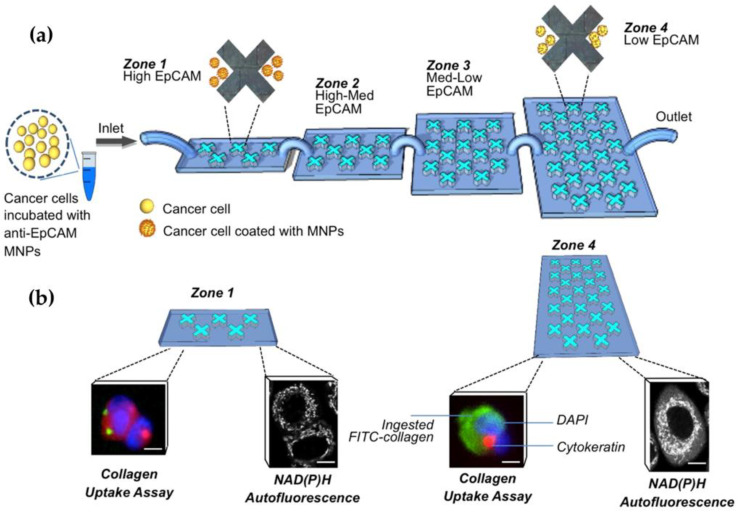
Phenotypic profiling of cancer cell subpopulations; (**a**) schematic showing the separation of cancer cells into four zones of a microfluidic device; (**b**) phenotypic analysis of isolated tumor cells [93].

Breast cancer is increasingly the most common cancer in women and its diagnosis is a broadly investigated topic currently [91,92]. Microfluidic cell sorting systems have the potential to increase the characterization of heterogeneous cell populations. This technology allows the separation of cells based on their surface marker expression level, adhesion capacity, transportability, and migration potential, respectively. Green et al. used nanoparticle intermediated sorting for phenotypically distinct breast cancer cell separation. They coated magnetic nanoparticles (MNPs) with antibodies against the epithelial cell adhesion molecule (EpCAM) using a microfluidic platform for the isolation process of cancer cells. They assessed the cell phenotypic properties using low EpCAM and high EpCAM by matrix-coated surfaces. Also, they tested and reported SKBR3 cells undergoing metabolic changes that induced hypoxia [93]. They demonstrated the capacity of this technology to the isolation of cancer cells that display different functional and biochemical phenotypes. The analyses of cellular subpopulations are undertaken by a microfluidic platform with functional assays (Figure 4).

The earliest stage of breast cancer is ductal carcinoma in situ (DCIS), whereby tumor cells remain inside the mammary duct during DCIS. In a study, a novel microfluidic system was developed that recapitulates the DCIS microenvironment. Here, a grown DCIS model cell line was embedded in a 3D hydrogel with mammary fibroblasts. Then the behavior of cells was followed by confocal microscopy and optical metabolic imaging. On the other hand, nuclear magnetic resonance spectroscopy (NMR) was used for metabolite profile studies. RT-qPCR was utilized for gene expression analyzes. In this study, results showed that the DCIS cell metabolism led to hypoxia and nutrient starvation, revealing an altered metabolism focused on glycolysis and other hypoxia-associated pathways [94].

**Figure 5:**
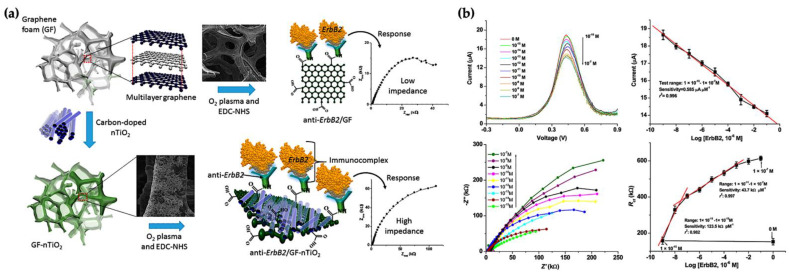
(**a**) Graphene foam (GF) and GF−nTiO2 electrodes; (**b**) DPV for different ErbB2 concentrations from 1.0 fM to 0.1 μM [95].

Azahar Ali et al. designed a femtomolar sensitivity and high-selectivity microfluidic immunosensor for real-time detection of epidermal growth factor receptor 2 (EGFR2 or ErbB2) proteins to quantify breast cancer biomarkers. They used a novel structured immuno-electrode made of porous hierarchical graphene foam (GF) modified with electrospun carbon-doped titanium dioxide nanofibers (nTiO_2_) as an electrochemical sensor electrode. nTiO_2_ is an ideal material due to perfect biocompatibility, high reaction kinetics, intrinsic surface defects, and excellent stability for proteins in electrochemical sensing applications. In this study, providing EDC–NHS chemistry covalently immobilized the antibody of ErbB2 (anti-ErbB2) on the GF–nTiO_2_ composite. To design a compact novel sensing system structure, the composite electrode was prepared to hang above the gold counter electrode in a microfluidic channel. The designed microfluidic platform had a broad concentration range of target ErbB2 antigen from 1.0 fM to 0.1 μM and from 0.1 pM to 0.1 μM, respectively. Many promising applications for the detection of chemical and biological species in the field of electrochemicals will reproduce from the combination of the porous GF–nTiO_2_ composite material onto microfluidic tools [95]. GF and GF−nTiO_2_ electrodes and response curves of the microfluidic immunosensor in detecting different ErbB2 concentrations are given in Figure 5a,b.

**Figure 6:**
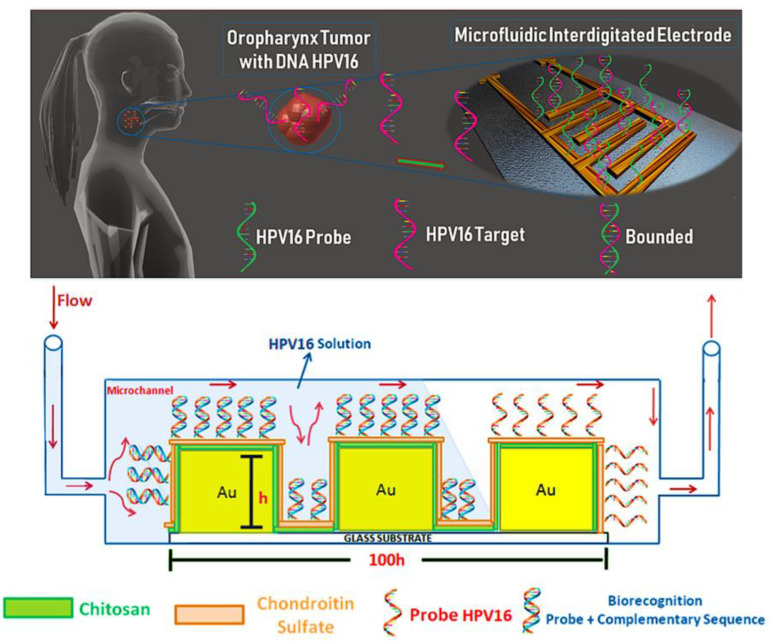
Schematic representation of the functionalization of the electrode and detection of HPV16 under continuous flow [98].

Head and neck squamous cell carcinomas of head and neck cancer display high rates of morbidity and mortality and are between the 10 most common human malignancies [96,97]. Detection of high-risk human papillomavirus (HPV) is crucial for head and neck cancer due to it being progressively regarded as a significant etiological factor. The present clinical tests are not performed routinely in public health systems owing to the limitations of the existing tests and high cost. In a study, a potential genosensor capable of detection of HPV16 at low concentrations and distinguishing was reported. The genosensor prepared by the microfluidic tool that had an active layer of an HPV16 capture DNA probe (cpHPV16) deposited onto a layer-by-layer polymer film of a chondroitin sulfate and chitosan. Impedance spectroscopy was used as the principle of detection. The limit of detection (LOD) was found to be 10.5 pM for complementary ssDNA HPV16 oligos (ssHPV16) [98]. The schematic image of the cpHPV16 probe is shown in Figure 6.

Oral cancer is a class of head and neck cancers. In another study, Zoupanou et al. developed a SMILE platform that is a cost-effective vehicle for early screening oral cancer. They reported that this platform has highly sensitive, selective, and easy-to-use properties. They also aim to reach many possible users and to prevent the disease. They employed PMMA substrates and the Mini-Mill/GX micro-milling machine with a 200 μm two-flute carbide micro end milling device. The modified EpCAM mouse monoclonal antibodies were immobilized on the surface of polymethyl methacrylate (PMMA) microchannels. The two diverse samples were prepared separately and contained 1 × 10^6^ cells/mL from the Jurkat line and 1 × 10^4^ cells/mL from the OECM line. The cell suspension was injected slowly through the serpentine channel with a flow rate of 7 μL/min. They also reported that the proposed platform may also be of excellent significance in the case of cancer cell research from other body fluids such as saliva [99].

The early sensing of ovarian cancer can raise the survival ratio of cancer patients notably. Aptamer-based optical and electrochemical biosensors integrated with microfluidic systems maintain to essentially advance the detection of molecular biomarkers of various detrimental diseases as quantitative including ovarian cancer and other kinds of solid tumor [100]. Tsai et al., designed and fabricated an integrated microfluidic chip that could make the entire circulating tumor cells (CTCs) isolation process. The 26 mm × 45 mm microfluidic chip was equipped with micropumps, micromixers, microvalves, and several chambers. They developed a novel integrated microfluidic chip capable of red blood cell (RBC) lysis, white blood cell (WBC) depletion, and ovarian cancer cell (OCC) capture via aptamer-coated magnetic beads in this study, which was able to detect OCCs vaccinated at concentrations of only 100 cells.ml^1^ in human blood fluids. The developed microfluidic chip was represented as a promising means of detecting low-abundance CTCs, specifically OCCs, from human blood samples and therefore could be a helpful vehicle for the early diagnosis of ovarian cancer. The designed cost-efficient and sensitive aptamer-based microfluidics sensing platform can be used as a reliable tool in the detection of ovarian cancer [101]. The smart nano-biomaterials are used in the development of aptamer-based microfluidic tools for the detection of ovarian cancer biomarkers, including CA-125 and CA-15 cancer antigens and, also, other types of cancer if suitable aptamers have been employed.

As microfluidic systems became a key technology for cancer diagnostics, the study of this area is increased. Microfluidic devices have been designed for the analysis of varied biomarkers. Developing low-cost sensitive systems to routinely measure the levels of multiple biomarkers from plasma, serum or whole blood could enable personalized medicine for cancer patients. 

## 4. Conclusions

Although there is a recent advancement in medical science with regards to developed diagnosis and pharmacological treatment protocols, there is a long way to go to reach a point where complex diseases including cancer will be accurately diagnosed and treated. Microfluidic tools have great potential as a new platform. Microfluidic tools have began to show their impact in biological research more than two decades ago. In recent years, there has been a great progress in the application of microfluidics in cancer research, diagnosis, and therapy. Cancer is one of the main causes of death worldwide. Cancer is a heterogeneous group of diseases that requires a multimodal paradigm to diagnose, apply, and treat. Microfluidic tools hold great promise in cancer diagnosis. Early cancer diagnosis results in better chances for real-time monitoring of treatment that will provide major impact and minor side effects. Studies have demonstrated that it serves as an emerging tool for understanding the biology of cancer and detection of cancer cells. Microfluidics are invaluable for cancer research owing to their high throughput, high sensitivity, low cost, consumption of less material, and improved spatio-temporal control. The physical laws on a sub-micro scale provide an advantage enabling the control of chemistry, biology, physics, and physiology at cellular level. In addition, microfluidic tools are portable, and they can be easily fabricated to be used as point of care diagnostics. These characteristics, in the age of coronavirus, are noteworthy. In the context of the pandemic, automated and low-cost devices can minimize interactions between patients and medical staff, given direct sampling from households and facilitating access to virus testing. The development of these devices can further reduce the chance of infection without affecting access to large-scale screening programs for cancer and other diseases.
